# Resistome Study in Aquatic Environments

**DOI:** 10.4014/jmb.2210.10044

**Published:** 2022-12-22

**Authors:** Hanseob Shin, Yongjin Kim, Seunggyun Han, Hor-Gil Hur

**Affiliations:** School of Earth Sciences and Environmental Engineering, Gwangju Institute of Science and Technology (GIST), Gwangju 61005, Republic of Korea

**Keywords:** Antibiotic resistance genes, antibiotic resistant bacteria, resistome

## Abstract

Since the first discovery of antibiotics, introduction of new antibiotics has been coupled with the occurrence of antibiotic resistant bacteria (ARB) and antibiotic resistance genes (ARGs). Rapid dissemination of ARB and ARGs in the aquatic environments has become a global concern. ARB and ARGs have been already disseminated in the aquatic environments via various routes. Main hosts of most of ARGs were found to belong to Gammaproteobacteria class, including clinically important potential pathogens. Transmission of ARGs also occurs by horizontal gene transfer (HGT) mechanisms between bacterial strains in the aquatic environments, resulting in ubiquity of ARGs. Thus, a few of ARGs and MGEs (*e.g.*, *strA*, *sul1*, *int1*) have been suggested as indicators for global comparability of contamination level in the aquatic environments. With ARB and ARGs contamination, the occurrence of critical pathogens has been globally issued due to their widespread in the aquatic environments. Thus, active surveillance systems have been launched worldwide. In this review, we described advancement of methodologies for ARGs detection, and occurrence of ARB and ARGs and their dissemination in the aquatic environments. Even though numerous studies have been conducted for ARB and ARGs, there is still no clear strategy to tackle antibiotic resistance (AR) in the aquatic environments. At least, for consistent surveillance, a strict framework should be established for further research in the aquatic environments.

## Introduction

Since the first serendipitous discovery of penicillin by Fleming in 1928 [1], a number of antibiotics have been found, synthesized and introduced for clinical purposes. Different classes of antibiotics such as aminoglycoside, chloramphenicol, glycopeptide, lipopeptide, macrolide, quinolone sulfonamide, and tetracycline have been introduced for clinical purposes [2]. Thus, bacterial strains have developed the ability to resist antibiotics. After release of antibiotic, vancomycin in 1958, vancomycin resistant *Enterococcus faecium* [3] and *Staphylococcus aureus* [4] were identified in 1988 and 2002, respectively. With introduction of methicillin, methicillin-resistant *S. aureus* was identified in 1960 [5]. In addition, *Klebsiella pneumoniae* carbapenemase (KPC)-producing *K. pneumoniae* emerged in 1996 after the approval of usage of imipenem in 1985 [6]. In 2017, priority pathogens were arranged by World Health Organization (WHO) where carbapenem-resistant *Acinetobacter baumannii* was selected as the highest priority of critical pathogens, followed by carbapenem-resistant *P. aeruginosa* and carbapenem-resistant *Enterobacteriaceae* family with resistance to the third generation of cephalosporin [7]. For surveillance of antibiotic resistance (AR), WHO has launched Global Antimicrobial Resistance Surveillance System (GLASS), in which more than 50 countries joined and shared comprehensive data on antibiotic resistant bacteria (ARB) and antibiotic resistance genes (ARGs). Also, WHO reported that 700,000 people have died from resistant infections, and more than 10 million would die in 2050 [8], which has been documented in United States [9].

Such warnings have triggered more numerous studies on AR in various environmental settings such as clinical settings, aquatic and agricultural environments, animals, and wastewater treatment plants (WWTPs). Those settings are interconnected as a complex web, forming one-health perspective. In the view of one health, ARB and ARGs could spread between humans, animals, and the environments. In particular, aquatic environments are prone to contamination from different sources [11] and thus provide favorable habitats for environmental bacteria to grow and exchange genetic materials [10]. Also, the aquatic environments could act for blooming of ARB and ARGs, and supply the water for public, animals or agricultural fields. It is thought to be a role of the aquatic environmnets as a “reservoir” for ARB and ARGs accumulation. Additionally, in aquatic environments, contaminant factors such as antibiotics, heavy metals and other components could serve as a selection factors to confer AR [12]. Additionally, non-antibiotic pharmaceuticals have been reported to promote HGT of ARGs through bacterial transformation [13]. For investigation of transmission of ARGs among environmental bacteria, various methods have been applied, and metagenomics could reveal the hosts of ARGs, overcoming non-cultivability of a majority of environmental bacteria [14].

This review provides the description of major ARGs occurrence in the aquatic environments, possible sources, transmission mechanisms, and factors for the bloom of ARGs. Frameworks to challenge the threat of ARB and ARGs against human health should be discussed in further research. Specifically, for globally consistent surveillance, standardized methodologies should be established in future research, which should be cost- and labor-effective.

## Advancement of Methodologies for ARGs Detection

**Fig. 1:**
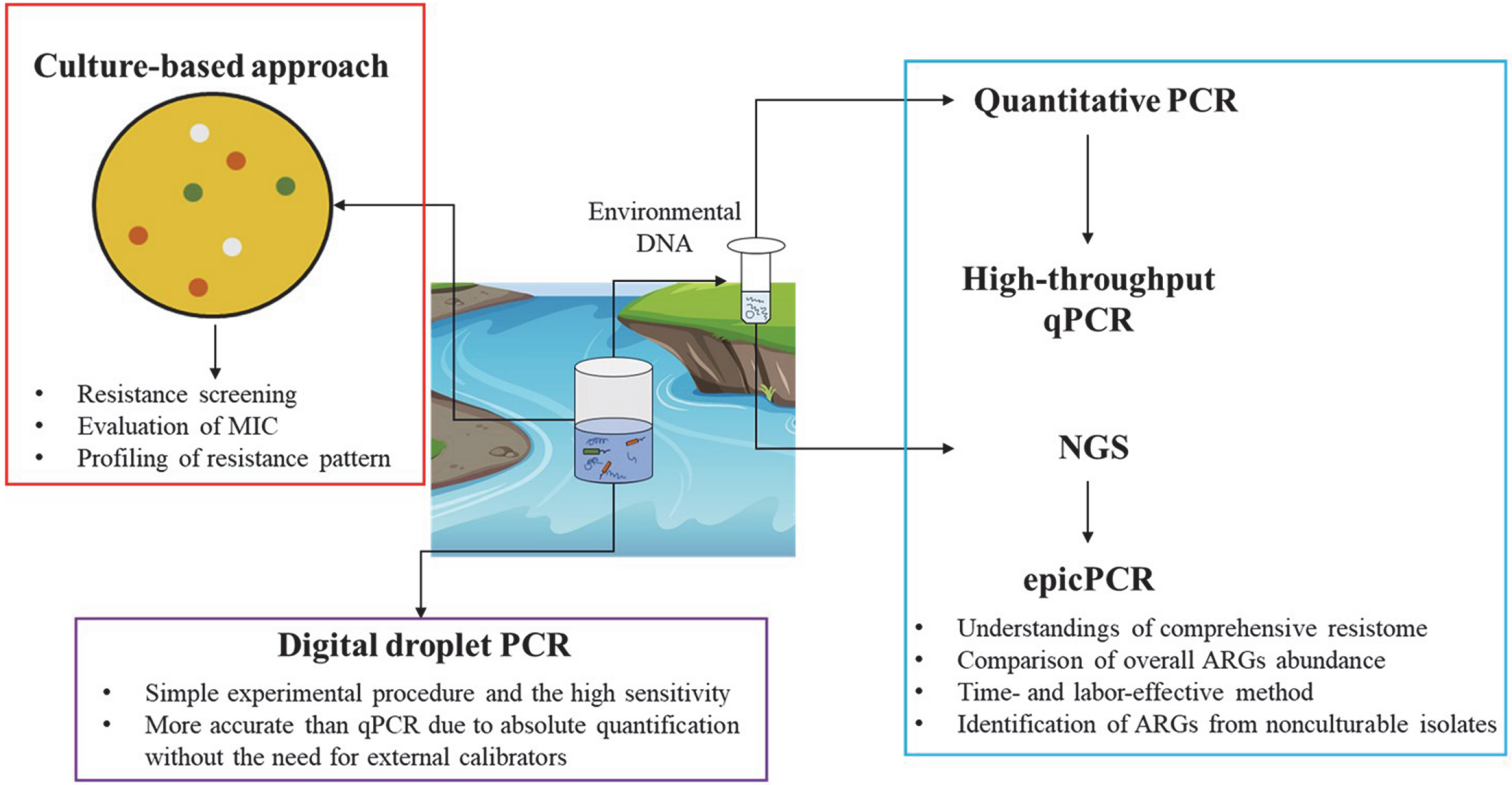
Advancement of methodologies for analyses of ARB and ARGs in aquatic environments.

There have been culture-dependent and independent approach tools, which can be applied for analysis of resistome in aquatic environments (Fig. 1). Culture-based methods have been used for practical risk assessment of antibiotic resistance in aquatic environments. Culture-based methods include isolation of bacteria, screening at a wide range of antibiotic concentrations, multiple resistance profile of the target organisms, and evaluation of minimal inhibition concentrations (MICs) of antibiotics. Researches have commonly aimed at human pathogenic bacteria such as *A. baumannii*, *Enterobacteriaceae* family, and *S. aureus*, which have been published as members of the critical pathogens [15]. Even though culture-based methods require much time, isolation, and identification of ARB and ARGs should not be underestimated due to the key understanding of phenotypic and genotypic characteristics. It can suggest the link between resistome in the environment and clinical settings.

PCR amplification of ARGs from ARB or environmental sample sources has been the most important method to determine the presence of resistance determinants. This method is a simple and fast way to detect target genes from bacteria or sample sources. However, it cannot yield quantity, for which qPCR could be applied for quantitative analyses of ARGs from environmental settings [16, 17]. The qPCR is one of the culture-independent methods to quantitatively analyze ARGs from cultivable and non-cultivable bacteria of environmental samples. The application of qPCR depends on use of primers to detect target ARGs and is difficult to cover all variants of ARGs with given primers. Thus, it is cost-, labor-, and time-consuming to quantitatively detect a wide range of classes and variants of ARGs.

A format for amplification of 384 ARGs in 5184 qPCR assays, including MGEs, were developed in 2012 [18]. The high throughput (HT) qPCR method has been widely used for a comprehensive profiling of ARGs in aquatic environments [19-23]. With newly discovered ARGs such as variants of *mcr*, a new primer set was published in 2018 [24], covering most of the classes of ARGs and MGEs. SmartChip instrument (Takara) has been commercially produced, which can quantitatively analyze 5184 assays per reaction using up to 384 pairs of primers. Even though it involves a wide range of ARG classes, amplification is limited to usage of primer sets. Even though this PCR-based approach amplifies a massive amount of ARGs, it still does not substitute for NGS-based screening for ARGs.

Recently, digital PCR (dPCR) has been developed, which can absolutely quantify the concentration of nucleic acids. It does not require calibration, which is different from qPCR-based approach. Droplet digital PCR (ddPCR) was developed from dPCR based on partitioning (to mimic limiting dilution) and Poisson statistics to overcome the qPCR limitations [25]. The ddPCR has capacity to generate more than thousands of partitioned droplets for quantification of target genes. It has already been applied to investigate ARGs [26, 27] and fecal indicator bacteria [28].

NGS includes whole-genome sequencing to identify genomic sequences of bacteria. It can provide the presence of ARGs, virulence determinants, genes associated with mobility, and typing information for bacterial characteristics. Center for Genomic Epidemiology (CGE) has provided services to identify acquired ARGs (ResFinder), pathogenicity (PathogenFinder), virulence factors (VirulenceFinder), and several methods to type bacterial isolates, by which global surveillance has been investigated. Even though genetic information of resistance determinants and associated MGEs could be organized, it cannot substitute phenotypic resistance which can be a guideline in clinical settings. Recently, long read sequencing (Oxford Nanopore, Pacific Biosciences Single Molecule Real-time (SMRT)) has appeared to identify ARGs, which can confer higher accuracy to track the host of a gene.

For sequencing-based comprehensive analyses of resistome, untargeted (shotgun) sequencing of all (meta) genomes has appeared [29]. Through shotgun metagenomics, pieces of gene fragments (reads) can be generated and assembled into contigs, which are computationally reconstructed for target functional genes or microbial genomes. Genomic sequences derived from metagenomics have been deposited in genome database such as NCBI (National Center for Biotechnology Information) or EMBL (European Molecular Biology Laboratory). Reconstruction of bacterial genomes by metagenomics enabled to reconstruct the metabolism of bacteria in global oceans [30]. Metagenomic approach has also facilitated the large-scale investigations of widespread presence of ARB and ARGs in aquatic environments [31, 32]. The sequences from metagenomics are annotated based on the database of ARGs such as Antibiotic Resistance Genes Database (ARDB, https://ardb.cbcb.umd.edu/) or The Comprehensive Antibiotic Resistance Database (CARD, https://card.mcmaster.ca/). It has expanded the coverage of ARGs detection and newly emerging variants, and enabled to identify the hosts of ARGs by reconstruction of bacterial genomes. For direct, easy and fast host identification, single cell-based approach, Emulsion, Paired Isolation and Concatenation PCR (epicPCR), has been developed [33]. In a bacterial single cell, genes of 16S rDNA and a target are amplified and fused into one fragment. In turn, the fragment is sequenced, and computationally processed to identify the gene and its host [33]. This method was performed to identify host of four genes in influents and effluent of WWTP [30] because profiling host is critical to determine the health risk to public. Nevertheless, it still cannot determine the phenotypic resistance pattern of bacterial isolates.

Culture-independent methods have been applied for comprehensive investigation of ARB and ARGs in aquatic environments. However, culture-dependent methods are also critical for risk assessment of bacterial isolates carrying ARGs. Culture-dependent approaches are mainly responsible for single isolate and practical parameters such as the minimum inhibitory concentrations (MICs) to determine clinical breakpoint. Culture-independent approaches contribute to the description, distribution, and dynamics of overall microbial community and ARGs in a sample. Thus, the decision should be dependent on the specific hypothesis of each research.

## Antibiotic Resistance in the Aquatic Environments

### Occurrence of ARGs and ARB in the Aquatic Environments

Introduction and application of antibiotics into human, livestock, and agriculture have employed an influence on environmental microbiome, with conferring antibiotic resistances genetically given by transferrable ARGs. Accordingly, various ARGs have been detected in diverse aquatic environments through several detection methods. In order to better understand the distribution and the diversity of ARGs in the aquatic environments, we manually screened and randomly selected related papers regardless of methodology and regions. Aminoglycoside, β-lactam, sulfonamide, and tetracycline, resistance genes have been mainly detected, which are described in this review (Table 1).

Aminoglycoside is a bactericidal drug grouped based on their chemical structures. Representatives include amikacin, gentamicin, streptomycin, and tobramycin, widely used for the treatment of infections of abdomen and urinary tract, and endocarditis caused by gram-negative bacteria, and certain gram-positive bacteria in combination with β-lactam or peptidoglycan [34]. Typical bacterial resistance mechanisms include modification of aminoglycoside, encoded by *aphA6* (aminoglycoside phosphotransferases), *aacC1* (aminoglycoside acetyltransferases), *aadB* and *aadA1* (aminoglycoside nucleotidyltransferases). Those genes have been documented to be equipped into plasmids and transposons [35, 36], suggesting the possibility to be widely disseminated in various host genera isolated from water environments. In the aquatic environments, various contaminants including antibiotics are present at low concentration by biological metabolisms [37]. The sub-inhibitory concentration of gentamycin could lead to resistance dispersion, by which aminoglycoside resistance determinants have been mainly documented from the potential pathogenic bacteria, including *A. baumannii*, *E. coli*, and *P. aeruginosa*.

β-Lactams belong to the group of most extensively used antibiotics for treatment of common bacterial infections. Resistance to β-lactam antibiotics is becoming problems in clinical settings because they could cover a wide range of bacterial infections by Gram-positive and -negative bacteria. Especially from *Enterobacteriaceae* family, a numerous of β-lactamases variants have been documented from various environmental settings [38-40]. The *bla* genes are classified into Class A, B, C, and D based on their amino acid sequences [41]. They are frequently associated with other ARGs on mobile genetic elements, escalating possibility of environmental spread [42, 43]. Resistance to β-lactams were also developed and selected after treatment processes with shift of relative abundance of Gammaproteobacteria [44]. It may suggest *bla* genes have been disseminated mainly in certain bacterial groups with selection pressure. From WWTPs, clinically important β-lactam resistant pathogens and extended-spectrum β-lactamase genes were released into the river water. Especially, biofilms were considered as a reservoir of wastewater-derived β-lactam resistant pathogens. It may herald clinically widely used β-lactams have expanded the bloom of resistome in the aquatic environments.

Sulfonamides are the first antibiotic class introduced into clinical settings for selective bacterial infections, targeting dihydropoteroate synthase responsible for reduction of dihydrofolate [45]. Mobile sulfonamide resistance genes were determined as three variants, *sul1*, *sul2* and *sul3*. Sulfonamide resistance genes have been documented as a class of the most dominant ARGs in the aquatic environments [16, 46]. Among the three genes, *sul1* gene has been detected in higher frequency, followed by *sul2* and *sul3* genes [47-49], linked with MGEs such as integrase integrons (*int1* and *int2*), insertion sequences (IS*26*), and wide range of incompatible plasmid groups (FII, FIB, I1, FIA, B/O, FIC, N, HI1 and X1) [47, 50, 51]. In the aquatic environments, *sul* genes could be horizontally transferred between different bacterial strains, aided by such various mobile genetic vehicles.

Tetracycline is well known and aging antibiotic to act against both Gram-positive and -negative bacteria. Tetracycline exhibits their effect by disturbing ribosomes for protein synthesis, and bacteria resist against their activity by expression of *tet* genes. The *tet* genes responsible for tetracycline resistance typically consist of *tetA*, *tetB*, *tetC*, *tetD*, and *tetE* (specific efflux pump for normally functional ribosomes) [52], and *tetM*, *tetO*, *tetS*, *tetT*, *tetQ* and *tetW* (ribosome protection from tetracyclines) [53]. Even though some of those genes are positioned on nonmobile plasmids or chromosomes without transferability [54], most of the *tet* genes have been found on broad host range plasmids [55]. The *tetM* gene was suspected to be on various plasmids and transposons, specifically Tn1545 in the aquatic environments of Vietnam [56]. In China, *tetA* and *tetB* were most frequently detected with 43% and 40% respectively from *Enterobacteriaceae* family in Pearl river [57]. Metagenomic analysis revealed *tetG* gene with *sul1* and *floR* genes were carried by *IS*91 family transposase, influenced by anthropogenic pollution [58]. The ability to move by such MGEs have already disseminated *tet* genes in the aquatic environments. In the aquatic environments, nonclinical isolates acquired *tet* genes by HGT mechanism, possibly acting as a reservoir [59].

The major hosts of ARGs studied in this review were found to belong to Proteobacteria, especially Gammaproteobacteria. Gammaproteobacteria is one of the dominant class in the aquatic environments and consists of clinically significant pathogenic bacteria such as Enterobacteriaceae, Moraxellaceae, Pseudomonadaceae, and *Vibrio*naceae. Due to the clinical relevance and potential pathogenicity of the several members of Gammaproteobacteria (*e.g.*, *Salmonella*, *Yersinia*, *Vibrio*, *P. aeruginosa*), they have been widely investigated and their vast genomic sequences have been deposited in genomic reference database. Gammaproteobacteria is thought to have exchanged and disseminated ARGs between the bacterial strains, and consequently enhance the bloom of ARG-carrying Gammaproteobacteria [60, 61]. Such selection is responsible for spread of ARGs mainly within Gammaproteobacteria [60, 61]. Even though genomic plasticity [62] and cargo genes [63] possibly provide clues for dissemination of ARGs within Gammaproteobacteria, it is required to further study on how several members of Gammaproteobacteria class become major hosts of ARGs.

### Source of ARB and ARGs in the Aquatic Environments

In natural environments, antibiotics have been produced by bacteria to kill other bacteria. However, acceleration of the evolution and spread of AR is responsible for other various contaminant factors from humans [64]/animals [65], inappropriate disposal of drugs [66], or wastewater from WWTPs [67], or run-off from agricultural environment or livestock by rainfall [68]. The distribution of ARB and ARGs have been investigated to examine human-related sources of ARB and ARGs [69-71]. From WWTPs, human-related ARB and ARGs have been discharged, possibly indicating WWTPs could be a source of contamination by anthropogenic activity [67]. ARGs, such as *ermF*, *ermT*, *sul1*, *sul2*, *tetB*, *tetG*, *tetX*, *qnrA*, *qnrB*, and *qnrS*, were frequently detected and suggested as indicators for wastewater due to the higher abundance near the effluent discharge in the receiving water [72]. WWTPs have been considered one of the host spots of ARB and ARGs because WWTPs receive contaminant from hospitals and households sweage, which could be important sources of ARB/ARGs and antibiotic residues [73]. Wastewater is one of the favorable habitats of bacteria due to the carbon sources, other various nutrients, and potential electron acceptors (such as oxygen, and nitrate). Proliferation of ARB and ARGs in WWTPs could influence on the ARB and ARG pollution in aquatic environments, especially receiving water. From WWTPs, enriched ARGs and ARB affect the abundance of ARGs and ARB even with reduction of AR after treatment process [67]. Although the removal rate of ARB was approximately 99%, significant amount of ARB and ARGs still remained in the treated sewages, being discharged into adjacent water bodies [74]. In addition, metagenomic analysis also revealed that *sul1* and *APH(3′′)-lb* were significantly more abundant in effluents [75]. Another study demonstrated that the ARGs evidently increased from upstream to downstream on the Han River, which were considered to result from anthropogenic activity [76]. In China, the abundance of ARGs were investigated and compared between pristine and anthropogenic activity-impacted areas [77]. Livestock production region is also considered as a source of antibiotics and ARB/ARGs of aquatic environments [78, 79]. With administration of antibiotics for growth and disease control of animals, ARB has developed their ability to resist against antibiotics in the animal bodies [80, 81]. Recently, a wide range of ARGs have been found in guts of animals with link to the introduction of antibiotics [18, 82]. In particular, sulfonamide and tetracycline resistance genes have been found as the most frequently detected ARGs by traditional quantitative PCR [83-85]. Introduction of metagenomics and high throughput qPCR, which can cover a broad range of classes of ARGs, showing presence of various ARGs in feces of animals [86, 87]. In China, mobile colistin resistance gene, *mcr-1*, was first isolated on plasmid pSHP45 of *E. coli* SHP45 from food animals. A previous study provided an evidence of impact of such sources, showing ARGs were significantly more abundant in the agriculture influenced river than the main stream [88].

## Horizontal Gene Transfer Mechanisms for ARGs Dissemination

**Fig. 2:**
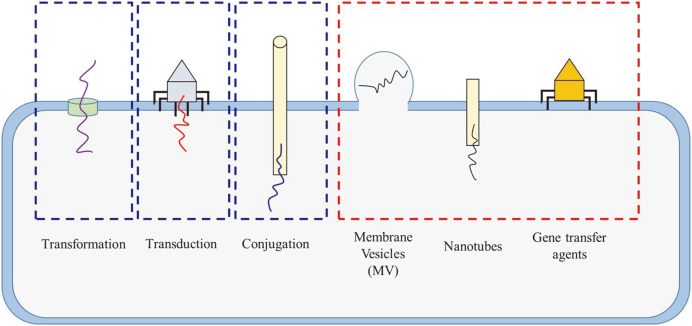
Mechanisms of horizontal gene transfer between bacterial strains. Blue and red boxes indicate well studied and newly emerging mechanisms, respectively.

The responsible events for dissemination of ARGs in environmental bacteria are mainly a result of horizontal gene transfer (HGT) of genetic materials from bacterial strains to different strains (Fig. 2). The transfer of ARGs is mediated by mobile genetic elements (MGEs) to confer the ability to travel between bacterial strains by association with insertion sequences (ISs) [89] or integrons [90, 91]. Then, the genes are arranged on MGEs such as plasmids, integrons, and ISs, which help to move between bacterial strains. The localized ARGs on MGEs could be transferred via HGT mechanisms between bacterial strains. Major mechanisms involved in HGT are followings: [1] conjugative transfer by mobile genetic elements, [2] transformation by free DNA, in the case of an environmentally induced competence such as the presence of calcium, and [3] transduction by bacteriophage. Recently, additional mechanisms have been revealed, being involved in HGT, such as [1] gene transfer agents (GTAs), [2] nanotubes between bacterial strains, and [3] membrane vesicles (MVs).

Via such mechanisms, vehicles for ARGs could travel between bacterial strains. Plasmids carry a number of genes for antibiotic and heavy metal resistance genes [92, 93]. Plasmids are classified into various incompatibility (Inc) types according to the inability for plasmids to coexist in the same bacterial cell [94]. Inc plasmids have been detected from various environments, carrying a wide range of ARGs. In addition, fused Inc plasmids were found to carry significantly higher number of ARGs than single Inc plasmids [95]. Indeed, a fused IncHI2/N and X3 plasmids from *E. coli* carries *bla*_NDM-5_ and 14 ARGs, respectively [36]. ARGs, including *bla*_TEM21_, *floR*, *dfr*, *qacE2*, *sul1*, *tetA*, *tetD* and *tetE* genes and a number of aminoglycoside resistance genes were carried by IncA/C plasmid of *Yersinia pestis* [96]. Plasmids isolated in the aquatic environments also showed high transfer frequencies to recipient bacterial cells with ARGs [97]. Thus, conjugative plasmids could play an important role in disseminating ARGs between different bacterial strains.

Transposable elements were defined as jumping genes more than 70 years ago due to the ability to ship DNA sequences on one location to another. Prokaryotic transposable elements (DNA transposon) are *Tn* family agents to act as vehicles of ARGs with ISs [98], helping to disseminate ARGs [45]. ISs are parts of transposable elements, moving within or between bacterial genomes employing recombination systems [99]. ISs also generate specific gene cassette such as that of *bla*_NDM_-*ble*_MBL_-*trpF*-*dsbD* identified as being bracketed by IS*3000*-IS*Aba125*-IS*5* and IS*26* in *E. coli* isolated from influent of a WWTP [36], the genetic context of which is similar to that from clinical sources [100, 101]. IS*26* (a member of IS*6*), in particular, grows in importance of carrying clinically important ARGs detected in clinical *Enterobacteriaceae* family in both chromosome and plasmids [102]. In river-lake system, metagenomic assembly revealed that the genomic islands of IS*91*-*sul2* and IS*91*-*aadA1*-*qacH*-*tetC* were frequently detected in river-lake system [103]. In addition, an IS element, IS*Apl1* (a member of IS*30* family) was reported to be involved in arrangement and spread of *mcr-1* (mobile colistin resistance) gene which is recently identified in *E. coli* isolate in China [104]. The location of IS*Apl1* downstream of *mcr-1* was responsible for mobilization by comprehensive analysis of sequences in GenBank [105]. Likewise, various types of ISs have been identified to contribute to dissemination of ARGs.

Integrons integrate diverse functional genes, and play a crucial role as a contributor of those genes such as ARGs. Integrons are composed of three elements: the gene, *intI*, for site-specific recombinase (integrase), the adjacent recombination site (*attI*) recognized by the integrase and the promoter (Pc) located upstream of the integration site. Integrons integrate gene cassettes consisting of various functional genes such as ARGs and heavy metal resistance genes [106]. Integrons can be classified based on their integrase sequences into three types: class 1, 2, and 3 integrons [107]. Currently, PCR-based approach has been utilized to recover and identify the gene cassettes of integron structures. In aquatic environments, *int1* gene has been identified as to be ubiquitous in high abundance, harboring several ARGs [88, 108]. Indeed, strong correlation between *int1* and a number of ARGs has been observed in aquatic environments [109-111]. For example, by comparative sequence analysis, clinically important ARG classes, such as aminoglycoside, β-lactam, chloramphenicol, sulfonamide, and trimethoprim, were found to constitute the gene cassettes associated with class 1 integron in *Aeromonas caviae*, *A. baumannii*, *S. enterica*, and *P. aeruginosa* [36]. Since such clinically important ARGs have been thought to be derived from anthropogenic activity, class 1 integron was also suggested as a proxy for anthropogenic pollution [112]. Besides, the presence of ARGs on class 2 integron structure was found in *Enterobacteriaceae* family in India, with *dfrA1*-*sat2*-*aadA1* the most frequently detected [113]. Class 3 integron was detected with gene cassettes carrying *bla*_OXA-256_ and *aac(6′)-Ib* variants in *Enterobacter cloacae* from hospital effluent, suggesting it has possibly participated in dissemination of ARGs in clinical and environmental settings [114].

## Contaminant Factors for ARB Blooming in Aquatic Environment

### Factors Interacting with Enrichment of ARB and ARGs

In the aquatic environments, bacteria frequently face various organic or inorganic chemicals including agents for selection pressure. Antibiotics enter the aquatic environments through direct discharge of wastewater [115, 116]. Antibiotic residues have been well documented for enrichment of ARB and ARGs as selection force in aquatic environments [117]. A large amount antibiotics such as ciprofloxacin and ofloxacin have been discharged from hospitals, showing strong correlation with corresponding ARGs [118], which may suggest the residues could promote proliferation of specific ARGs. In the aquatic environments, fluoroquinolone, sulfamethoxazole, and tetracycline antibiotics were most frequently detected exhibiting correlation with the number of resistant *E. coli* strains [119, 120], with dynamic change of microbial composition toward harboring phenotypic or genotypic AR. Antibiotics in the aquatic environments could expedite the HGT between different bacterial strains [121, 122]. In addition, heavy-metals also can promote the plasmid-mediated horizontal transfer of ARGs between different bacterial strains [123]. Toxicity of heavy-metals could lead to the production of reactive oxygen species, which contributes to the damage of DNA in bacterial cells [124, 125]. Against contamination by toxic heavy metals such as As, Cu and Pb, environmental bacteria present phenotypic and genotypic resistance [126]. The co-selection of heavy metals was exhibited together with antibiotics [12], expanding bacterial resistome [127]. Aquatic environments collect antibiotics and heavy metals from various sources, and hence, the ability to transfer ARGs of environmental bacteria via conjugation are studied in such water bodies [11].

## Priority Pathogens in River Water

In 2017, a list of priority pathogenic bacteria has been published for development of new antibiotics [136]. Carbapenem-resistant *A. baumannii*, carbapenem-resistant *P. aeruginosa*, and carbapenem-resistant Enterobacteriaceae were selected as members of the most critical pathogens. Vancomycin-resistant *Enterococcus faecium* (VRE), vancomycin- and methicillin-resistant *S. aureus* (VRSA and MRSA), clarithromycin-resistant *Helicobacter pylori*, fluoroquinolone-resistant *Campylobacter*, fluoroquinolone-resistant *Salmonella* spp., fluoroquinolone- and the 3^rd^ generation cephalosporin-resistant *Neisseria gonorrhoeae* belonged to the groups of high priority pathogens. The occurrence of these pathogens could cause severe and intractable bacterial infections even with the use of a wide range of antibiotics. Especially, pathogenic bacteria have been frequently found in urban river because it receives anthropogenic sewages containing ARB and ARGs [137]. *Clostridium* and *Bacillus* were found as main human pathogenic bacteria, exhibiting positive correlation with ARGs [138]. In the Niger river, three pathogenic bacteria, 32 of *S. aureus*, 64 of *Salmonella* spp., and 82 of *E. coli* were isolated among 319 bacterial isolates, suggesting this river was responsible for high risk source of pathogenic ARB dissemination [139]. Metagenomic assembly also identified potential pathogenic bacteria such as *S. pneumoniae*, *E. coli*, and *A. baumannii* in the aquatic environments [140]. Worldwide occurrence of human pathogenic bacteria in the aquatic environments indicates the potential risk to human health.

## Concluding Marks

Development of new antibiotics has driven the occurrence of potential pathogenic ARB and dissemination of ARGs in clinical and environmental settings. Urban WWTPs receive sewages from hospitals, pharmaceutical facilities, livestock and households and the treated wastewater discharged into the aquatic environment. The receiving water act as a reservoir for ARB and ARGs to spread, evolve, and transfer. The river waters are the source of recreation, irrigation and drinking water, which are the major route of dissemination of ARB and ARGs from animals to humans. It has been suggested one-health ecosystem, and several studies on monitoring of ARB and ARGs have been conducted in those environmental settings. However, there is no clear strategies for mitigation of the presence of AR in the ecosystems. Thus, it is urgent to establish the framework to reduce AR from WWTPs and in the aquatic environments to prevent the spread of AR.

Investigation of the abundance of ARGs is important in the aquatic environments because it serves as a reservoir for dissemination of ARGs by diverse MGEs, on which integron integrase genes are equipped with ARGs. The most crucial thing is that ARGs on MGEs could be transferred from non-clinical isolates (environmental bacteria) to clinical isolates (potential pathogenic bacteria, originated from clinical settings). Several studies revealed that clinically critical pathogenic bacteria, possibly derived from clinical settings, have been found in the aquatic environments worldwide. It may result from ARGs acquisition of environmental bacteria from human/animals derived pathogenic bacteria or HGT of ARGs between clinical and environmental bacteria. However, it has already occurred for a long time, and now, surveillance is important for determination of contamination level in the aquatic environments. For consistent worldwide surveillance, clearly standardized methodologies should be established, such as indicator genes for cost- and labor-effective experiments, tools for quantitative analysis of targets, and the way to collect and process the water samples. Some of genes, including *int1*, have been documented for global comparability of contamination level. Using those genes, standardized and simple methods should be performed for the fair surveillance, the establishment of strategies and how to administrate the aquatic environments.

## Figures and Tables

**Table 1:** The major ARG variants investigated in different habitats of the aquatic environments from 16 countries.

Country	Source	ARGs	ARB	Reference
Belgium	River	*bla*_TEM_, *qnrS*, *sul1*, *sul2*, *tetW*, *tetO*	NA	[141]
Brazil	River	*bla*_TEM_, *ermB*, *qnrS*, *sul1*	NA	[142]
China	River	*bla*PSE, *bla*CTX-M, *ermA*, *ermB*, *qnrA*, *qnrS*, *sul1*, *sul2*, *tetW*, *tetA*	*Actinobacteria*, *Patescibacteria*, *Proteobacteria*	[134]
China	River	24 ARGs	*Acinetobacter*, *Caulobacteraceae*, *Cetobacterium*, *Lactobacillaceae*	[143]
China	River	*sul1*, *sul2*, *tetA*, *tetC*	NA	[144]
China	River	*aacC4*, *bla*_ampC_, *bla*_OXA-1_, *bla*_TEM-1_, *ermB*, *mphA*, *qnrS*, *strA*, *sul1*, *sul2*, *tetC*, *tetW*,	NA	[145]
China	River	212 ARGs (including *bla*_TEM_, *sul1*, *sul2*, *tetA*, *tetB*, *tetC*, *tetG*, *tetM*, *tetO*, *tetW*, *tetX*)	NA	[77]
China	DWTP	28 ARGs	*Aeromonas salmonicida*, *Pseudomonas aeruginosa*	[146]
China	WWTP	71 ARGs to aminoglycoside, tetracycline, and MLS	*Anaerolineaceae*, *Comamonadaceae*, *Rhizomicrobium*, *Saprospiraceae*	[147]
China	WWTP	*bla*_TEM_, *bla*_NDM-1_, *bla*_CTX-M-32_, *mcr-1*, *sul1*	NA	[148]
China	WWTP	*qnrC*, *qnrD*, *sul1*, *sul2*, *sul3*, *tetA*, *tetB*, *tetE*, *tetW*, *tetM*, tetZ	NA	[149]
China	WWTP	*intI1*, *sul1*, *sul2*, *tetO*, *tetW*, *tetQ*	NA	[150]
Ethiopia	WWTP	NA	*Citrobacter* spp., *E. coli*, *Klebsiella* spp., *Pseudomonas* spp., *S. aureus*	[151]
Finland	WWTP	*bla*_OXA-58_, *int1*, *tetM*	NA	[152]
Finland, Estonia	WWTP	*bla*_oxa-58_, *bla*_shv-34_, *bla*_ctx-m_, *sul1*, *sul2*, *tetM*, *tetC*	NA	[153]
India	River	*bla*_CTX-M1_, *bla*_CTX-M9_, *bla*_VIM_, *bla*_NDM_, *qnrS*, *qnrB*, *sul1*, *sul2*	*E. coli*	[119]
Netherlands	WWTP, River	*ermB*, *sul1*, *sul2*, *tetW*	NA	[154]
Nigeria	River	NA	*Bacillus* spp., *Micrococcus* spp., *Pseudomonas* spp., *Staphylococcus*, *Streptococcus*	[155]
Poland	River	*aac(6')-Ib-cr*, *bla*_CTX-M_, *bla*_OXA_, *bla*_SHV_, *bla*_TEM_, *ermS*, *linA*, *sul1*, *tet(A)*, *tet(B)*, *tet(C)*, *tet(D)*, *tet(E)*	NA	[156]
Poland	WWTP	*aac(6')-Ib-cr*, *bla*_SHV_, *bla*_TEM_, *sul1*, *tetA*	*E. coli*	[157]
Saudi Arabia	WWTP	*tetH*, *tetO*, *tetQ*, *tetW*, *tetZ*	*A. hydrophila*, *Enterobacter* spp., *Enterococcus*, *P. aeruginosa*	[158]
Singapore	WWTP	15 ARGs (including *bla*_KPC_, *bla*_NDM_, *bla*_SHV_, *ermB*, *intI1*, *sul1*, *tetO*)	*Aeromonas*, *Enterobacteriaceae*, *Pseudomonas*	[159]
South Korea	River	*bla*_CTX-M_, *bla*_OXA_, *bla*_SHV_, *bla*_TEM_	*E. coli*	[160]
South Korea	WWTP, River	*bla*_NDM-5_, *bla*_NDM-9_	*E. coli*, K. variicola	[161]
South Korea	Ocean	*aac(60)-Ib-cr*, *bla*_CTX_, *bla*_SHV_, *bla*_TEM_, *ermC*, *floR*, *int1*, *oqxA*, *qnrD*, *qnrS*, *sul1*, *sul2*, *tetA*, *tetB*, *tetBP*, *tetD*, *tetE*, *tetG*, tetH, *tetM*, *tetQ*, *tetX*, *tetZ*	*Bacteroidetes*, *Chloroplast*, *Cyanobacteria*, *Marinomonas*, *Proteobacteria*, *Verrucomicrobia*, *Vibrio*	[162]
Spain	WWTP, River	*bla*_TEM_, *ermB*, *qnrS*, *sul1*, *tetW*	NA	[118]
Spain	WWTP	*bla*_CTX-M_, *bla*_SHV_, *bla*_TEM_, *erm(B)*, *qnrS*, *sul(I)*, *sul(II)*, *tet(O)*, *tet(W)*	*Acinetobacter*, *Aeromonas*, *Exiguobacterium*, *Piscinibacter*, *Pseudohodoferax*, *Pseudomonas*	[163]
Switzerland	River	*aadA*, *bla*_CTX-M_, *bla*_NDM_, *bla*_SHV_, *bla*_TEM_	*E. coli*, *Enterococcus* spp., *Pseudomonas* spp.	[164]
Switzerland	WWTP	*qnrA*, *sul1*, *sul2*, *tet(B)*, *tet(M)*, *tet(W)*	NA	[165]
USA	River	*sul1*, *sul2*, *tetO*, *tetW*	NA	[166]

*NA; Not applicable

## References

[1] Fleming A (1929). On the antibacterial action of cultures of a penicillium, with special reference to their use in the isolation of *B. influenzæ*. Br. J. Exp. Pathol..

[2] Davies J, Davies D (2010). Origins and evolution of antibiotic resistance. Microbiol. Mol. Biol. Rev..

[3] Yesim C, Pamela F, Glen MC (2000). Vancomycin-resistant Enterococci. Clin. Microbiol. Rev..

[4] Centers for Disease Control and Prevention (CDC) (2002). *Staphylococcus aureus* resistant to vancomycin-United States 2002. MMWR. Morb. Mortal. Wkly. Rep..

[5] Jevons MP (1961). January. "Celbenin"-resistant Staphylococci. Br. Med. J..

[6] Yigit H, Queenan AM, Anderson GJ, Domenech-Sanchez A, Biddle JW, Steward CD (2001). Novel carbapenem-hydrolyzing beta-lactamase, KPC-1, from a carbapenem-resistant strain of *Klebsiella pneumoniae*. Antimicrob. Agents Chemother..

[7] Tacconelli E, Magrini N, Kahlmeter G, Singh N (2017). Global priority list of antibiotic-resistant bacteria to guide research, discovery, and development of new antibiotics. World Heal. Organ.

[8] Mancuso G, Midiri A, Gerace E, Biondo C (2021). Bacterial antibiotic resistance: the most critical pathogens. Pathogens.

[9] John P, Holdren, Eric S (2014). Reort to the president on combating antibiotic resistance.

[10] Bengtsson-Palme J, Kristiansson E, Larsson DGJ (2018). Environmental factors influencing the development and spread of antibiotic resistance. FEMS Microbiol. Rev..

[11] Hooban B, Joyce A, Fitzhenry K, Chique C, Morris D (2020). The role of the natural aquatic environment in the dissemination of extended spectrum beta-lactamase and carbapenemase encoding genes: a scoping review. Water Res..

[12] Li L-G, Xia Y, Zhang T (2017). Co-occurrence of antibiotic and metal resistance genes revealed in complete genome collection. ISME J..

[13] Wang Y, Lu J, Engelstädter J, Zhang S, Ding P, Mao L (2020). Non-antibiotic pharmaceuticals enhance the transmission of exogenous antibiotic resistance genes through bacterial transformation. ISME J..

[14] Amos GCA, Zhang L, Hawkey PM, Gaze WH, Wellington EM (2014). Functional metagenomic analysis reveals rivers are a reservoir for diverse antibiotic resistance genes. Vet. Microbiol..

[15] Asokan GV, Ramadhan T, Ahmed E, Sanad H (2019). WHO global priority pathogens list: a bibliometric analysis of medline-PubMed for knowledge mobilization to infection prevention and control practices in bahrain. Oman Med. J..

[16] Jiang L, Hu X, Xu T, Zhang H, Sheng D, Yin D (2013). Prevalence of antibiotic resistance genes and their relationship with antibiotics in the Huangpu River and the drinking water sources, Shanghai, China. Sci. Total Environ..

[17] Uta B, Hans-Henno D, Neus A-GM, Miquel Sde M, Valter T, Caterina L (2009). Quantitative PCR monitoring of antibiotic resistance genes and bacterial pathogens in three European artificial groundwater recharge systems. Appl. Environ. Microbiol..

[18] Looft T, Johnson TA, Allen HK, Bayles DO, Alt DP, Stedtfeld RD (2012). In-feed antibiotic effects on the swine intestinal microbiome. Proc. Natl. Acad. Sci. USA.

[19] George PBL, Rossi F, St-Germain M-W, Amato P, Badard T, Bergeron MG (2022). Antimicrobial resistance in the environment: towards elucidating the roles of bioaerosols in transmission and detection of antibacterial resistance genes. Antibiotics.

[20] Muziasari WI, Pärnänen K, Johnson TA, Lyra C, Karkman A, Stedtfeld RD (2016). Aquaculture changes the profile of antibiotic resistance and mobile genetic element associated genes in Baltic Sea sediments. FEMS Microbiol. Ecol..

[21] Karkman A, Johnson TA, Lyra C, Stedtfeld RD, Tamminen M, Tiedje JM (2016). High-throughput quantification of antibiotic resistance genes from an urban wastewater treatment plant. FEMS Microbiol. Ecol..

[22] Fernanda PA, Liu S, Yuan T, Ramalingam B, Lu J, Sekar R (2022). Diversity and abundance of antibiotic resistance genes and their relationship with nutrients and land use of the inflow rivers of Taihu Lake. Front. Microbiol..

[23] Muurinen J, Stedtfeld R, Karkman A, Pärnänen K, Tiedje J, Virta M (2017). Influence of manure application on the environmental resistome under finnish agricultural practice with restricted antibiotic use. Environ. Sci. Technol..

[24] Stedtfeld RD, Guo X, Stedtfeld TM, Sheng H, Williams MR, Hauschild K (2018). Primer set 2.0 for highly parallel qPCR array targeting antibiotic resistance genes and mobile genetic elements. FEMS Microbiol. Ecol..

[25] Huggett JF, Foy CA, Benes V, Emslie K, Garson JA, Haynes R (2013). The digital MIQE guidelines: minimum information for publication of quantitative digital PCR experiments. Clin. Chem..

[26] Wang X, Gu J, Gao H, Qian X, Li H (2018). Abundances of clinically relevant antibiotic resistance genes and bacterial community diversity in the weihe river, China. Int. J. Environ. Res. Public Health..

[27] Cavé L, Brothier E, Abrouk D, Bouda PS, Hien E, Nazaret S (2016). Efficiency and sensitivity of the digital droplet PCR for the quantification of antibiotic resistance genes in soils and organic residues. Appl. Microbiol. Biotechnol..

[28] Cao Y, Raith MR, Griffith JF (2015). Droplet digital PCR for simultaneous quantification of general and human-associated fecal indicators for water quality assessment. Water Res..

[29] Quince C, Walker AW, Simpson JT, Loman NJ, Segata N (2017). Shotgun metagenomics, from sampling to analysis. Nat. Biotechnol..

[30] Tully BJ, Graham ED, Heidelberg JF (2018). The reconstruction of 2,631 draft metagenome-assembled genomes from the global oceans. Sci. Data.

[31] Cui G, Liu Z, Xu W, Gao Y, Yang S, Grossart H-P (2022). Metagenomic exploration of antibiotic resistance genes and their hosts in aquaculture waters of the semi-closed Dongshan Bay (China). Sci. Total Environ..

[32] Bai Y, Ruan X, Li R, Zhang Y, Wang Z (2022). Metagenomics-based antibiotic resistance genes diversity and prevalence risk revealed by pathogenic bacterial host in Taihu Lake, China. Environ. Geochem. Health.

[33] Spencer SJ, Tamminen M V, Preheim SP, Guo MT, Briggs AW, Brito IL (2016). Massively parallel sequencing of single cells by epicPCR links functional genes with phylogenetic markers. ISME J..

[34] Gonzalez LS, Spencer JP (1998). Aminoglycosides: a practical review. Am. Fam. Physician.

[35] Lin M-F, Liou M-L, Tu C-C, Yeh H-W, Lan C-Y (2013). Molecular epidemiology of integron-associated antimicrobial gene cassettes in the clinical isolates of *Acinetobacter baumannii* from northern Taiwan. Ann. Lab. Med..

[36] Shin H, Kim Y, Han D, Hur H (2021). Emergence of high level carbapenem and extensively drug resistant *Escherichia coli* ST746 producing NDM-5 in influent of wastewater treatment plant, Seoul, South Korea. Front. Microbiol..

[37] Cairns J, Ruokolainen L, Hultman J, Tamminen M, Virta M, Hiltunen T (2018). Ecology determines how low antibiotic concentration impacts community composition and horizontal transfer of resistance genes. Commun. Biol..

[38] Carey AM, Capik SF, Giebel S, Nickodem C, Piñeiro JM, Scott HM (2022). Prevalence and profiles of antibiotic resistance genes mph(A) and qnrB in extended-spectrum Beta-Lactamase (ESBL)-producing *Escherichia coli* isolated from dairy calf feces. Microorganism.

[39] Legese MH, Asrat D, Aseffa A, Hasan B, Mihret A, Swedberg G (2022). Molecular epidemiology of extended-spectrum betalactamase and AmpC producing enterobacteriaceae among sepsis patients in Ethiopia: a prospective multicenter study. Antbiotics.

[40] Islam MS, Sobur MA, Rahman S, Ballah FM, Ievy S, Siddique MP (2022). Detection of blaTEM, blaCTX-M, blaCMY, and blaSHV genes among extended-spectrum beta-lactamase-producing *Escherichia coli* isolated from migratory birds travelling to Bangladesh. Microb. Ecol..

[41] Bush K, Jacoby GA (2010). Updated functional classification of beta-lactamases. Antimicrob. Agents Chemother..

[42] Weldhagen GF (2004). Integrons and β-lactamases-a novel perspective on resistance. Int. J. Antimicrob. Agents.

[43] Schlüter A, Szczepanowski R, Pühler A, Top EM (2007). Genomics of IncP-1 antibiotic resistance plasmids isolated from wastewater treatment plants provides evidence for a widely accessible drug resistance gene pool. FEMS Microbiol. Rev..

[44] Stachurová T, Piková H, Bartas M, Semerád J, Svobodová K, Malachová K (2021). Beta-lactam resistance development during the treatment processes of municipal wastewater treatment plants. Chemosphere.

[45] Alekshun MN, Levy SB (2007). Molecular mechanisms of antibacterial multidrug resistance. Cell.

[46] Pruden A, Pei R, Storteboom H, Carlson KH (2006). Antibiotic resistance genes as emerging contaminants: studies in Northern Colorado. Environ. Sci. Technol..

[47] Wu S, Dalsgaard A, Hammerum AM, Porsbo LJ, Jensen LB (2010). Prevalence and characterization of plasmids carrying sulfonamide resistance genes among *Escherichia coli* from pigs, pig carcasses and human. Acta Vet. Scand..

[48] Byrne-Bailey KG, Gaze WH, Kay P, Boxall ABA, Hawkey PM, Wellington EMH (2009). Prevalence of sulfonamide resistance genes in bacterial isolates from manured agricultural soils and pig slurry in the United Kingdom. Antimicrob. Agents Chemother..

[49] Gosia K Kozak, David L Pearl, Julia Parkman, Richard J Reid-Smith, Anne Deckert, Patrick Boerlin (2009). Distribution of sulfonamide resistance genes in *Escherichia coli* and *Salmonella* Isolates from swine and chickens at abattoirs in Ontario and Québec, Canada. Appl. Environ. Microbiol..

[50] Chen B, Liang X, Nie X, Huang X, Zou S, Li X (2015). The role of class I integrons in the dissemination of sulfonamide resistance genes in the Pearl River and Pearl River Estuary, South China. J. Hazard. Mater..

[51] Vincent P, Patrick B (2003). A new sulfonamide resistance gene (sul3) in *Escherichia coli* is widespread in the pig population of Switzerland. Antimicrob. Agents Chemother..

[52] Roberts MC (2002). Resistance to tetracycline, macrolide-lincosamide-streptogramin, trimethoprim, and sulfonamide drug classes. Mol. Biotechnol..

[53] Connell SR, Tracz DM, Nierhaus KH, Taylor DE (2003). Ribosomal protection proteins and their mechanism of tetracycline resistance. Antimicrob. Agents Chemother..

[54] Roberts MC (2007). Genetic mobility and distribution of tetracycline resistance determinants. Ciba Found. Symp..

[55] Zhang XX, Zhang T, Fang HHP (2009). Antibiotic resistance genes in water environment. Appl. Microbiol. Biotechnol..

[56] Kobayashi T, Suehiro F, Cach Tuyen B, Suzuki S (2007). Distribution and diversity of tetracycline resistance genes encoding ribosomal protection proteins in Mekong river sediments in Vietnam. FEMS Microbiol. Ecol..

[57] Tao R, Ying G-G, Su H-C, Zhou H-W, Sidhu JPS (2010). Detection of antibiotic resistance and tetracycline resistance genes in Enterobacteriaceae isolated from the Pearl rivers in South China. Environ. Pollut..

[58] Guan Y, Jia J, Fan X, Li K, Wang Z (2022). Anthropogenic impacts on antibiotic resistance genes and their hosts from pristine to urban river using metagenomic and binning approaches. Aquat. Toxicol..

[59] Perewari DO, Otokunefor K, Agbagwa OE (2022). Tetracycline-resistant genes in *Escherichia coli* from clinical and nonclinical sources in Rivers State, Nigeria. Int. J. Microbiol..

[60] Marti E, Jofre J, Balcazar JL (2013). Prevalence of antibiotic resistance genes and bacterial community composition in a river nfluenced by a wastewater treatment plant. PLoS One.

[61] Shin H, Kim Y, Raza S, Unno T, Ryu S-H, Hur H-G (2022). Dynamics of genotypic and phenotypic antibiotic resistance in a conventional wastewater treatment plant in 2 years. Front. Microbiol..

[62] Kloesges T, Popa O, Martin W, Dagan T (2011). Networks of gene sharing among 329 proteobacterial genomes reveal differences in lateral gene transfer frequency at different phylogenetic depths research article. Mol. Biol. Evol..

[63] Benler S, Faure G, Altae-Tran H, Shmakov S, Zheng F, Koonin E (2021). Cargo genes of Tn7-like transposons comprise an enormous diversity of defense systems, mobile genetic elements, and antibiotic resistance genes. mBio.

[64] Alfredo T, Brian A, CTT (2012). Selective pressure of antibiotic pollution on bacteria of importance to public health. Environ. Health Perspect..

[65] Gao F-Z, Zou H-Y, Wu D-L, Chen S, He L-Y, Zhang M (2020). Swine farming elevated the proliferation of acinetobacter with the prevalence of antibiotic resistance genes in the groundwater. Environ. Int..

[66] Azam M, Kumar V, Siddiqui K, Jan AT, Sabir JSM, Rather IA (2020). Pharmaceutical disposal facilitates the mobilization of resistance determinants among microbiota of polluted environment. Saudi Pharm. J..

[67] Buri I, Kuchta P, Mol A, David V, Rul M, Lochman J (2021). Antibiotic resistance in wastewater and its impact on a receiving river? a case study of WWTP Brno-Modřice, Czech Republic. Water.

[68] Kuchta SL, Cessna AJ (2009). Fate of lincomycin in snowmelt runoff from manure-amended pasture. Chemosphere.

[69] Kim S, Aga DS (2007). Potential ecological and human health impacts of antibiotics and antibiotic-resistant bacteria from wastewater treatment plants. J. Toxicol. Environ. Health. B. Crit. Rev..

[70] Durso LM, Miller DN, Wienhold BJ (2012). Distribution and quantification of antibiotic resistant genes and bacteria across agricultural and non-agricultural metagenomes. PLoS One.

[71] Schwartz T, Kohnen W, Jansen B, Obst U (2003). Detection of antibiotic-resistant bacteria and their resistance genes in wastewater, surface water, and drinking water biofilms. FEMS Microbiol. Ecol..

[72] Zhang Y, Wang J, Lu J, Wu J (2020). Antibiotic resistance genes might serve as new indicators for wastewater contamination of coastal waters: spatial distribution and source apportionment of antibiotic resistance genes in a coastal bay. Ecol. Indic..

[73] Deguenon E, Dougnon V, Houssou VMC, Gbotche E, Ahoyo RA, Fabiyi K (2022). Hospital effluents as sources of antibiotics residues, resistant bacteria and heavy metals in Benin. SN Appl. Sci..

[74] Korzeniewska E, Korzeniewska A, Harnisz M (2013). Ecotoxicology and environmental safety antibiotic resistant *Escherichia coli* in hospital and municipal sewage and their emission to the environment. Ecotoxicol. Environ. Saf..

[75] Raza S, Shin H, Hur H, Unno T, Shin H, Hur H (2021). Higher abundance of core antimicrobial resistant genes in effluent from wastewater treatment plants. Water Res..

[76] Lee K, Kim D, Lee D, Kim Y, Bu J, Cha J (2020). Mobile resistome of human gut and pathogen drives anthropogenic bloom of antibiotic resistance. Microbiome.

[77] Ouyang WY, Huang FY, Zhao Y, Li H, Su JQ (2015). Increased levels of antibiotic resistance in urban stream of Jiulongjiang River, China. Appl. Microbiol. Biotechnol..

[78] S Koike, I G Krapac, H D Oliver, A C Yannarell, JC Chee-Sanford, RI Aminov (2007). Monitoring and source tracking of tetracycline resistance genes in lagoons and groundwater adjacent to swine production facilities over a 3-year period. Appl. Environ. Microbiol..

[79] Zhang X, Li Y, Liu B, Wang J, Feng C, Gao M (2014). Prevalence of veterinary antibiotics and antibiotic- resistant *Escherichia coli* in the surface water of a livestock production region in Northern China. PLoS One.

[80] Heuer H, Schmitt H, Smalla K (2011). Antibiotic resistance gene spread due to manure application on agricultural fields. Curr. Opin. Microbiol..

[81] Marti R, Scott A, Tien Y-C, Murray R, Sabourin L, Zhang Y (2013). Impact of manure fertilization on the abundance of antibiotic-resistant bacteria and frequency of detection of antibiotic resistance genes in soil and on vegetables at harvest. Appl. Environ. Microbiol..

[82] Zhao Y, Su J-Q, An X-L, Huang F-Y, Rensing C, Brandt KK (2018). Feed additives shift gut microbiota and enrich antibiotic resistance in swine gut. Sci. Total Environ..

[83] Pavelquesi SLS, de Oliveira Ferreira ACA, Rodrigues ARM, de Souza Silva CM, Orsi DC, da Silva ICR (2021). Presence of tetracycline and sulfonamide resistance genes in *Salmonella* spp. literature review. Antibiotics.

[84] L Qing, D Qigen, H Jian, W Hongjun, C Jingdu (2022). Profiles of tetracycline resistance genes in paddy soils with three different organic fertilizer applications. Environ. Pollut..

[85] Olonitola OS, Fahrenfeld N, Pruden A (2015). Antibiotic resistance profiles among mesophilic aerobic bacteria in Nigerian chicken litter and associated antibiotic resistance genes1 1The authors express appreciation to the council for international exchange of scholars/institute of international educ. Poult. Sci..

[86] Xiao L, Estellé J, Kiilerich P, Ramayo-Caldas Y, Xia Z, Feng Q (2016). A reference gene catalogue of the pig gut microbiome. Nat. Microbiol..

[87] Wang H, Sangwan N, Li H-Y, Su J-Q, Oyang W-Y, Zhang Z-J (2017). The antibiotic resistome of swine manure is significantly altered by association with the Musca domestica larvae gut microbiome. ISME J..

[88] Lu L, He Y, Peng C, Wen X, Ye Y, Ren D (2022). Dispersal of antibiotic resistance genes in an agricultural influenced multibranch river network. Sci. Total Environ..

[89] Razavi M, Kristiansson E, Flach C-F, Larsson DGJ, LaPara TM (2020). The association between insertion sequences and antibiotic resistance genes. mSphere.

[90] Razavi M, Marathe NP, Gillings MR, Flach C-F, Kristiansson E, Joakim Larsson DG (2017). Discovery of the fourth mobile sulfonamide resistance gene. Microbiome.

[91] Gillings M, Boucher Y, Labbate M, Holmes A, Krishnan S, Holley M (2008). The evolution of class 1 integrons and the rise of antibiotic resistance. J. Bacteriol..

[92] Bennett PM (2008). Plasmid encoded antibiotic resistance: acquisition and transfer of antibiotic resistance genes in bacteria. Br. J. Pharmacol..

[93] Partridge SR, Tsafnat G, Coiera E, Iredell JR (2009). Gene cassettes and cassette arrays in mobile resistance integrons. FEMS Microbiol. Rev..

[94] Couturier M, Bex F, Bergquist PL, Maas WK (1988). Identification and classification of bacterial plasmids. Microbiol. Rev..

[95] Li Z, Li Z, Peng Y, Lu X, Kan B (2022). Trans-regional and cross-host spread of *mcr*-carrying plasmids revealed by complete plasmid sequences-44 countries, 1998-2020. China CDC Wkly..

[96] Jing-Cao P, Rong Y, Hao-Qiu W, Hai-Qing X, Wei Z, Xin-Fen Y (2008). *Vibrio cholerae* O139 multiple-drug resistance mediated by *Yersinia pestis* pIP1202-like conjugative plasmids. Antimicrob. Agents Chemother..

[97] Dang B, Mao D, Xu Y, Luo Y (2017). Conjugative multi-resistant plasmids in Haihe river and their impacts on the abundance and spatial distribution of antibiotic resistance genes. Water Res..

[98] Babakhani S, Oloomi M (2018). Transposons: the agents of antibiotic resistance in bacteria. J. Basic Microbiol..

[99] Siguier P, Gourbeyre E, Chandler M (2014). Bacterial insertion sequences: their genomic impact and diversity. FEMS Microbiol. Rev..

[100] Yuan Y, Li Y, Wang G, Li C, Chang Y F, Chen W (2019). blaNDM-5 carried by a hypervirulent *Klebsiella pneumoniae* with sequence type 29. Antimicrob. Resist. Infect. Control.

[101] Zhu YQ, Zhao JY, Xu C, Zhao H, Jia N, Li YN (2016). Identification of an NDM-5-producing *Escherichia coli* sequence Type 167 in a neonatal patient in China. Sci. Rep..

[102] Varani A, He S, Siguier P, Ross K, Chandler M (2021). The IS6 family, a clinically important group of insertion sequences including IS*26*. Mob. DNA.

[103] Chen H, Li Y, Sun W, Song L, Zuo R, Teng Y (2020). Characterization and source identification of antibiotic resistance genes in the sediments of an interconnected river-lake system. Environ. Int..

[104] Liu YY, Wang Y, Walsh TR, Yi LX, Zhang R, Spencer J (2016). Emergence of plasmid-mediated colistin resistance mechanism MCR-1 in animals and human beings in China: a microbiological and molecular biological study. Lancet Infect. Dis..

[105] Snesrud E, McGann P, Chandler M (2018). The birth and demise of the IS *Apl1-mcr-1-IS-Apl1* composite transposon: the vehicle for transferable colistin resistance. mBio.

[106] Stalder T, Barraud O, Casellas M, Dagot C, Ploy M-C (2012). Integron involvement in environmental spread of antibiotic resistance. Front. Microbiol..

[107] R GM (2014). Integrons: past, present, and future. Microbiol. Mol. Biol. Rev..

[108] Hubeny J, Korzeniewska E, Buta-Hubeny M, Zieliński W, Rolbiecki D, Harnisz M (2022). Characterization of carbapenem resistance in environmental samples and *Acinetobacter* spp. isolates from wastewater and river water in Poland. Sci. Total Environ..

[109] Nguyen TN, Kasuga I, Liu M, Katayama H (2019). Occurrence of antibiotic resistance genes as emerging contaminants in watersheds of Tama River and lake Kasumigaura in Japan. IOP Conf. Ser. Earth Environ. Sci..

[110] Agramont J, Gutiérrez-Cortez S, Joffré E, Sjöling Å, Calderon Toledo C (2020). Fecal pollution drives antibiotic resistance and class 1 integron abundance in aquatic environments of the bolivian andes impacted by mining and wastewater. Microorganism.

[111] Zheng W, Huyan J, Tian Z, Zhang Y, Wen X (2020). Clinical class 1 integron-integrase gene - A promising indicator to monitor the abundance and elimination of antibiotic resistance genes in an urban wastewater treatment plant. Environ. Int..

[112] Gillings MR, Gaze WH, Pruden A, Smalla K, Tiedje JM, Ku J (2014). Using the class 1 integron-integrase gene as a proxy for anthropogenic pollution. ISME J..

[113] Singha P, Chanda DD, Maurya AP, Paul D, Chakravarty A, Bhattacharjee A (2016). Distribution of class II integrons and their contribution to antibiotic resistance within *Enterobacteriaceae* family in India. Indian J. Med. Microbiol..

[114] Barraud O, Casellas M, Dagot C, Ploy M-C (2013). An antibiotic-resistant class 3 integron in an *Enterobacter cloacae* isolate from hospital effluent. Clin. Microbiol. Infect..

[115] Heberer T (2002). Occurrence, fate, and removal of pharmaceutical residues in the aquatic environment: a review of recent research data. Toxicol. Lett..

[116] Kemper N (2008). Veterinary antibiotics in the aquatic and terrestrial environment. Ecol. Indic..

[117] (2017). Environmental Chemicals, the Human Microbiome, and Health Risk.

[118] Rodriguez-Mozaz S, Chamorro S, Marti E, Huerta B, Gros M, Sànchez-Melsió A (2015). Occurrence of antibiotics and antibiotic resistance genes in hospital and urban wastewaters and their impact on the receiving river. Water Res..

[119] Hanna N, Purohit M, Diwan V, Chandran SP, Riggi E, Parashar V (2020). Monitoring of water quality, antibiotic residues, and antibiotic-resistant *Escherichia coli* in the Kshipra river in India over a 3-year period. Int. J. Environ. Res. Public Health.

[120] Zhang Q, Jia A, Wan Y, Liu H, Wang K, Peng H (2014). Occurrences of three classes of antibiotics in a natural river basin: association with antibiotic-resistant *Escherichia coli*. Environ. Sci. Technol..

[121] Lu J, Wang Y, Li J, Mao L, Nguyen SH, Duarte T, Guo J (2018). Triclosan at environmentally relevant concentrations promotes horizontal transfer of multidrug resistance genes within and across bacterial genera. Environ. Int..

[122] Wang Y, Lu J, Mao L, Li J, Yuan Z, Bond PL (2019). Antiepileptic drug carbamazepine promotes horizontal transfer of plasmidborne multi-antibiotic resistance genes within and across bacterial genera. ISME J..

[123] Qiu Z, Yu Y, Chen Z, Jin M, Yang D, Zhao Z (2012). Nanoalumina promotes the horizontal transfer of multiresistance genes mediated by plasmids across genera. Proc. Natl. Acad. Sci. USA.

[124] Szivák I, Behra R, Sigg L (2009). Metal-induced reactive oxygen species production in *Chlamydomonas reinhardtii* (Chlorophyceae). J. Phycol..

[125] Caitlin L Williams, Heather M Neu, Jeremy J Gilbreath, Sarah LJ Michel, Daniel V Zurawski, Scott Merrell (2016). Copper resistance of the emerging pathogen *Acinetobacter baumannii*. Appl. Environ. Microbiol..

[126] Carrasco A, Armario P, Chamber MA, Palomares AJ, Caviedes A, Lo R (2005). Isolation and characterisation of symbiotically effective Rhizobium resistant to arsenic and heavy metals after the toxic spill at the Aznalcóllar pyrite mine. Soil Biol. Biochem..

[127] Berg J, Thorsen MK, Holm PE, Jensen J, Nybroe O, Brandt KK (2010). Cu exposure under field conditions coselects for antibiotic resistance as determined by a novel cultivation-independent bacterial community tolerance assay. Environ. Sci. Technol..

[128] Wilson LA, Rogers Van Katwyk S, Fafard P, Viens AM, Hoffman SJ (2020). Lessons learned from COVID-19 for the post-antibiotic future. Global. Health.

[129] Nieuwlaat R, Mbuagbaw L, Mertz D, Burrows LL, Bowdish DME, Moja L (2021). Coronavirus disease 2019 and antimicrobial resistance: parallel and interacting health emergencies. Clin. Infect. Dis..

[130] Ashiru-Oredope D, Kerr F, Hughes S, Urch J, Lanzman M, Yau T (2021). Assessing the impact of COVID-19 on antimicrobial stewardship activities/programs in the United Kingdom. Antibiotics.

[131] Clancy CJ, Nguyen MH (2020). Coronavirus disease 2019, superinfections, and antimicrobial development: what can we expect?. Clin. Infect. Dis..

[132] Contou D, Claudinon A, Pajot O, Micaëlo M, Longuet Flandre P, Dubert M (2020). Bacterial and viral co-infections in patients with severe SARS-CoV-2 pneumonia admitted to a French ICU. Ann. Intensive Care.

[133] Tiri B, Sensi E, Marsiliani V, Cantarini M, Priante G, Vernelli C (2020). Antimicrobial stewardship program, COVID-19, and infection control: spread of carbapenem-resistant *Klebsiella Pneumoniae* colonization in ICU COVID-19 patients. What did not work?. J. Clin. Med..

[134] Zhang L, Zhang C, Lian K, Ke D, Xie T, Liu C (2021). River restoration changes distributions of antibiotics, antibiotic resistance genes, and microbial community. Sci. Total Environ..

[135] Kuroda K, Li C, Dhangar K, Kumar M (2021). Predicted occurrence, ecotoxicological risk and environmentally acquired resistance of antiviral drugs associated with COVID-19 in environmental waters. Sci. Total Environ..

[136] Harbarth S, Kahlmeter G, Kluytmans J, Mendelson M, Hospital GS, Town C, Burkert (2017). Global priority list of antibiotic-resistant bacteria to guide research, discovery, and development of new antibiotics.

[137] Fang T, Wang H, Cui Q, Rogers M, Dong P (2018). Diversity of potential antibiotic-resistant bacterial pathogens and the effect of suspended particles on the spread of antibiotic resistance in urban recreational water. Water Res..

[138] Zhao Z, Li C, Jiang L, Wu D, Shi H, Xiao G, Kang X (2022). Occurrence and distribution of antibiotic resistant bacteria and genes in the Fuhe urban river and its driving mechanism. Sci. Total Environ..

[139] Koumaré Y, Babana AH, Bah A, Kassogué A, Dao S, Diallo K, Faradji F (2022). Bacteria isolated from Niger River water in Bamako showed multi-resistance to antibiotics. MOJ Biol. Med..

[140] Wang L, Zhu M, Li Y, Zhao Z, Hu T (2022). Deterministic assembly process dominates bacterial antibiotic resistome in wastewater effluents receiving river. Environ. Sci. Pollut. Res. Int..

